# Xp 11.2 translocation renal carcinoma in young adults; recently classified distinct subtype

**DOI:** 10.2478/raon-2013-0077

**Published:** 2014-04-25

**Authors:** Andrej Kmetec, Jera Jeruc

**Affiliations:** 1 Urologic Clinical Department, University Medical Centre Ljubljana, Ljubljana, Slovenia; 2 Institute of Pathology, Medical Faculty, University of Ljubljana, Ljubljana, Slovenia

**Keywords:** renal tumours, translocation renal cell carcinoma, histology

## Abstract

**Background:**

XP11.2 renal translocation carcinomas are often encountered in paediatric group of patients where they are believed to be rather indolent. They are rare but more aggressive in young adults. They are slow growing, sometimes without characteristic symptoms and their biologic behaviour is uncertain.

**Case report:**

We report two cases of this type of tumour in Slovenian young adult males with long and unusual history. Tumours were confirmed imunohistologically by positive reaction for CD10, P504S and TFE3.

**Conclusions:**

According to the indications in the literature prognosis of these tumours in young adults depends upon the stage. It seems that cysts, haematomas and necrosis around the kidney are often encountered in these tumours. In advanced stage with lymph nodes involvement or distant metastases, the prognosis is poor. Surgery seems to be basic mode of therapy.

## Introduction

Renal cell carcinoma (RCC) represents 2.9% of all carcinomas in Slovenia. The crude incidence was increasing in males from 10.6/100000 and females 6.1/100000 in the period 1993–97 to 20.4/100000 in males and 10.5/100000 in females during the period 2005–2009.^1^–^3^ Cancer originates in the epithelium of the proximal convoluted tubule filtering the blood and it accounts for more than 90% of all renal malignancies occurring in adults. The 2004 World Health Organisation (WHO) classification distinguishes three main histologic types: clear cell, papillary and chromophobe renal cell carcinoma.^4^ Lately, the use of new imunohistologic and molecular techniques, has recognised some rare, uncommon unclassified types of tumours, *e.g.* Bellini duct carcinoma, medullary carcinoma, Xp11.2 translocation carcinoma, mucinous tubular and spindle cell carcinoma.^5^,^6^ These new entities comprise only 10–15% of renal tumours, but they have important implication on the outcome. Yet for some subtypes the prognosis and the optimal way of treatment is still not well defined.^6^

Xp11.2 translocation carcinoma has been recently recognised as a distinct subtype of renal carcinoma. Xp11.2 renal cell carcinomas are defined by at lest six different translocations involving Xp11.2 chromosome, all of which result in a gene fusion involving the TFE3 (transcription factor E3) gene.^7^–^16^ This subtype of renal cell tumour occurs predominantly in the paediatric group where it accounts for 20–40% of paediatric renal cell carcinoma. It is very rare in adults, the incidence has been reported to be 1–1.6% of all renal tumours, but its actual incidence remains underestimated.^8^,^9^ Meta-analyses of cases in the literature found that 50% or even 65% of patients with Xp11.2 translocation renal cell carcinoma presented with high-stage tumours, namely in stage III and IV.^9^ Classification is the same as for all renal cell tumours (Table 1). Complete surgical removal of the tumour mass including the kidney is the preferred therapy in patients with lower stage tumours. In patients with metastatic or relapsed carcinoma targeted agents are used such as sunitinib and mTOR inhibitors, while chemotherapy is not effective.^9^ Malouf *et al.* in his study concluded that Xp11 translation renal cell carcinoma targeted therapy achieve objective responses and prolonged progression-free survival.^9^ Prognosis of patients in higher stages is poor, most of them die within a year after the surgery, while the prognosis of patients with low stage disease is variable because the exact biologic behaviour of tumours and impact of current treatment modalities remains uncertain.^10^ Prognosis depends also on the age: in children tumour can be rather indolent, but in patients aged 16 or older Xp11.2 translocation carcinoma has a more aggressive clinical course.^11^,^12^

## Cases presentation

We present the first two cases of Xp11.2 translocation renal cell carcinomas confirmed in Slovenia in two young males admitted to the urological department in the period of three months. The first one, aged 27, was accepted urgently due to an unbearable pain and a palpable tumour in the abdominal and lumbar region. CT scan revealed a huge solid tumour mass measuring 7.6 × 8.2 × 8.1 cm located in the lower and lateral part of right kidney with metastatic tumours of similar size in the retroperitoneal region, over and under the vena cava, between the aorta and the vena cava extending up to the liver and down to the aortal bifurcation.

**FIGURES 1, 2.**
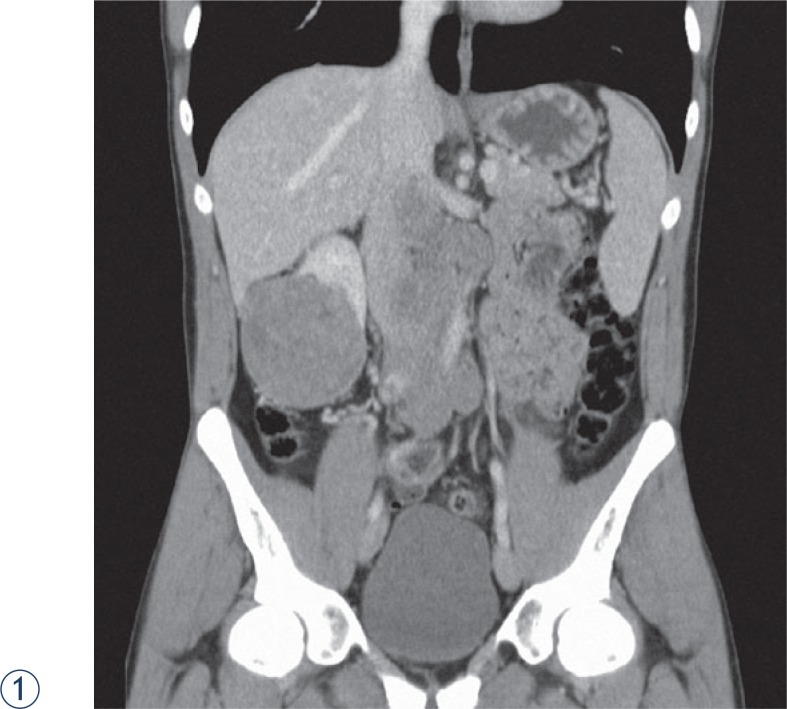

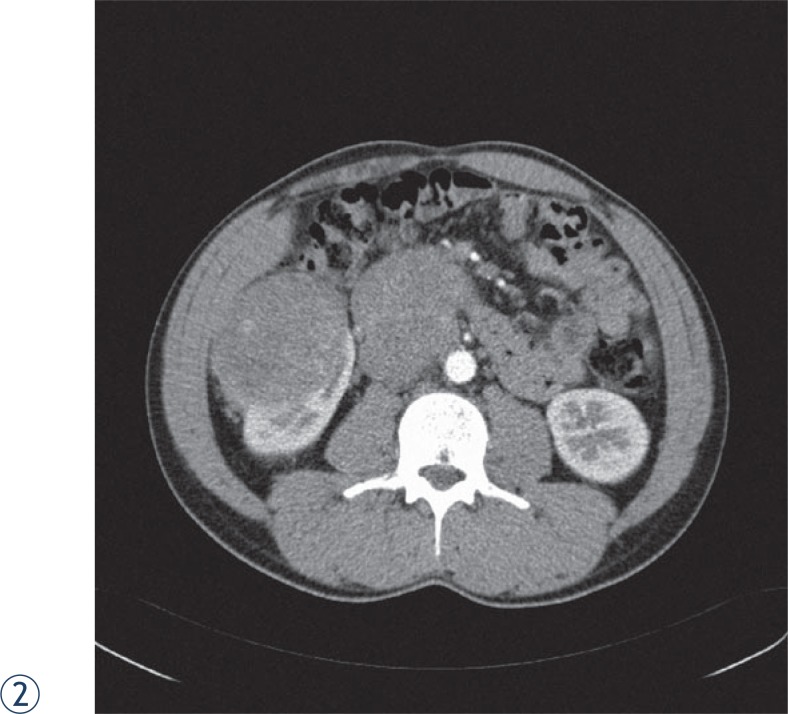
Abdominal CT scan of 27 year adult male.

Four years prior the last hospitalisation, he was admitted to hospital also due to a pain in the lumbar region. At the time CT scan and ultrasound examination revealed a septal haematoma with a thick wall measuring 10 cm in diameter on the anterior side of the right kidney and an angiomyolipoma-like change on the lower pole of the same kidney measuring 3.5 × 2.5 cm. The cause of the haematoma was not clearly identified, bleeding from angiomyolipoma or trauma was suspected. Furthermore, CT scan showed a solid mass near the kidney haematoma that was not further investigated or been even overlooked. Months later, the ultrasound investigation confirmed that haematoma decreased and showed the persistent angiomyolipoma of the same size without any solid mass around the kidney. Because the patient was asymptomatic, he did not attend regular controls until lumbar and abdominal pain re-emerged after four years. Tumour biopsy verified a solid renal tumour. Due to the persistent pain and haematuria, embolization of the kidney and tumour mass was performed. Using a transabdominal surgical approach we managed to remove the kidney with the tumour and the haematoma and well delineated retroperitoneal metastases along vena cava. Tumour burden was removed radically, but eight months later local recurrence and distant lymph nodes were established. The patient refused additional surgical intervention and he preferred treatment at department for oncology. He was given sunitinib as the first line therapy, but no objective response was achieved. Treatment changed to mTOR inhibitor (everolimus), also without any objective response. Despite specific therapy the disease progressed and later only symptomatic treatment was introduced. A year after the surgery the patient died because of massive cancer involvement (Figures 1 and 2).

**FIGURE 3, 4.**
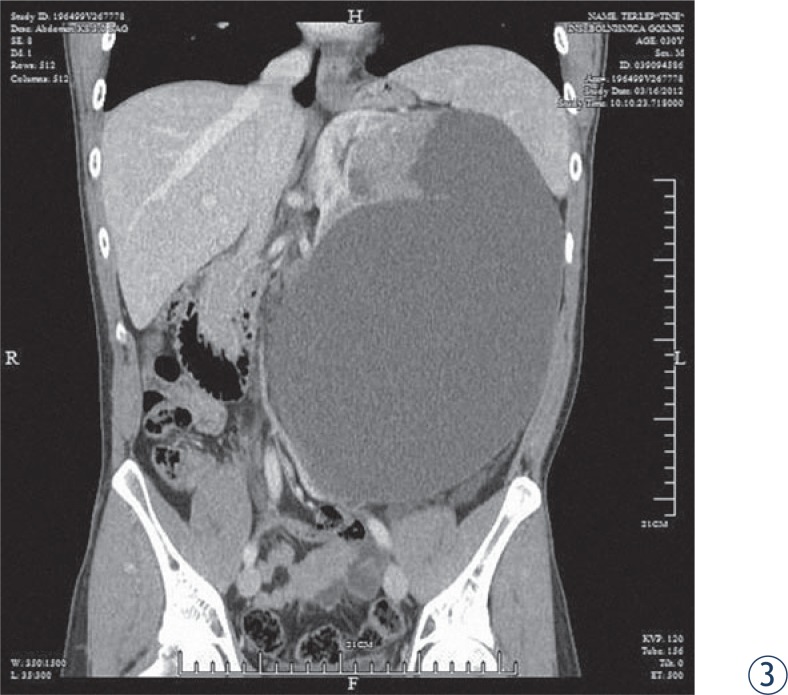

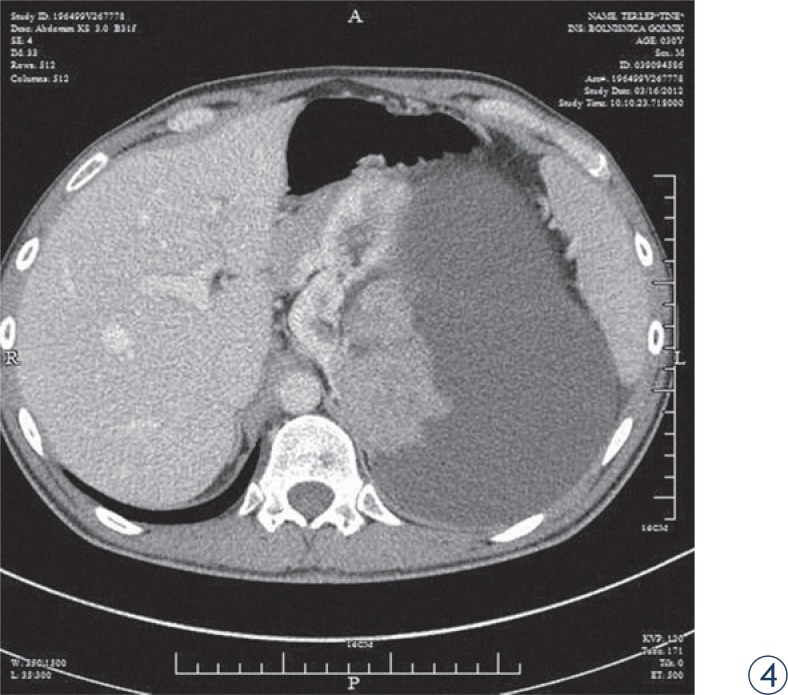
CT scan of the 31 year adult male with cystic mass.

The second patient, aged 31, experienced sudden respiratory distress and was admitted to the pulmonary department where pulmonary embolism was confirmed and treated. The CT scan revealed a huge cystic formation measuring 28 × 21 × 16 cm embracing the left kidney with a solid mass near compressed kidney. The formation distended to the abdominal wall. As the patient thought he has been gaining weight, has been practising slimming diets for over a year. Otherwise the patient felt no pain; his only complaint was shortness of breath. Prior the surgery cava filter was inserted into the lower vena cava. Surgical procedure was done using lumbar approach, a huge cystic cavity was isolated and 3.5 l of cloudy liquid was evacuated. A compressed kidney with adjacent tumour was removed along with the entire tumour mass. Lymph nodes were negative and tumour extension over cystic margins was not detected. Ten months after surgery the patient is still asymptomatic (Figures 3 and 4)

### Histology

**FIGURE 5.**
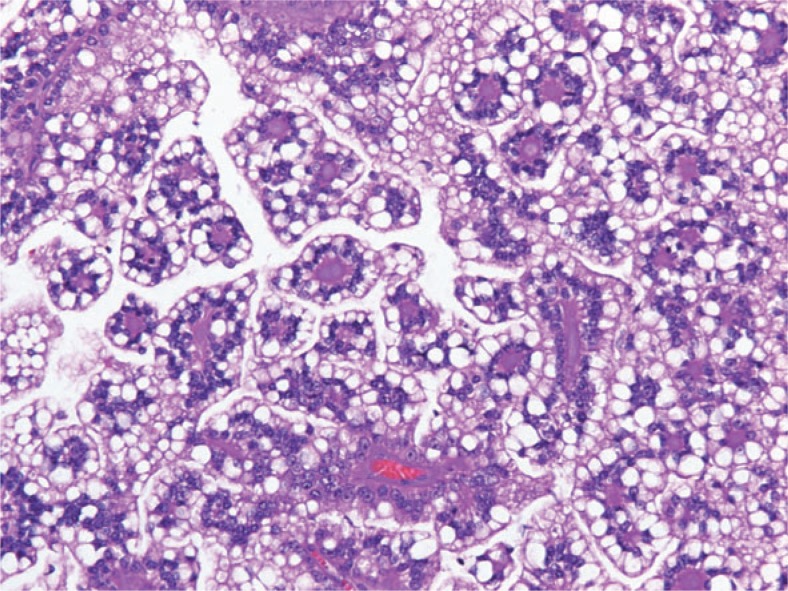
Translocation renal cell carcinoma composed of clear cells with voluminous cytoplasm and distinct cell borders showing typical papillary architecture and hyalinised fibrovascular cords.

In the first case the kidney contained a well circumscribed solid, yellowish tumour with a central haemorrhage and necrosis, measuring 6.5 × 5.3 cm and grossly confined by the renal capsule. The uninvolved renal parenchyma was pale with dark brown spots, consistent with postembolization changes. There were five additional tumour nodules, weighing 292 g in total, all well circumscribed, some with adherent fat. On the cut surface, the tumours appeared multilobulated, soft, tan-pink, with tissue organised into papillary structures. Histologically the tumour was composed of were extensive areas of necrosis and haemorrhage surrounded by hemosiderin-laden macrophages. The neoplastic cells were diffusely immunoreactive for RCC antigen and racemase, focally for CD10 and negative for CK7, EMA, HMB45 and Melan A (Figure 5)

**FIGURE 6.**
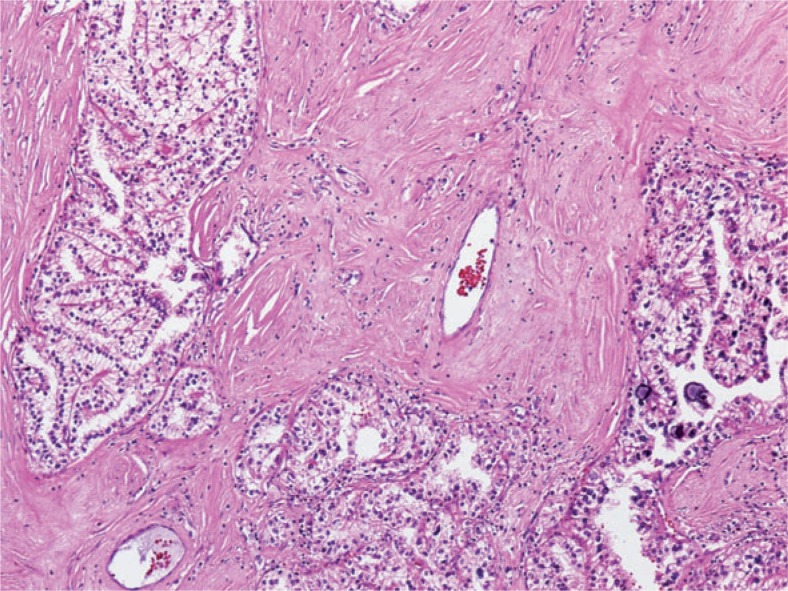
Translocation renal cell carcinoma composed of clear cells arranged in tubule-alveolar pattern laying in hyalinised stroma. There are some psammoma bodies in the lower right corner.

In the second case the tumour was cystic, the solid part of the yellowish-gray tumour with necrotic and haemorrhagic areas measured 9.4 × 7.1 cm. Microscopic examination revealed a clear cell papillary tumour with abundant hyalinised stroma. Tumour cells showed partially clear and partially eosinophilic cytoplasm and enlarged hyperchromatic nuclei. There were numerous psammoma bodies, larger calcifications and some hyaline globules. The neoplastic cells were diffusely immunoreactive for RCC antigen, racemase, CD 10 and vimentin and negative for CK7 and EMA. Angiomyolipoma was not found in the resected specimen (Figure 6)

**FIGURE 7.**
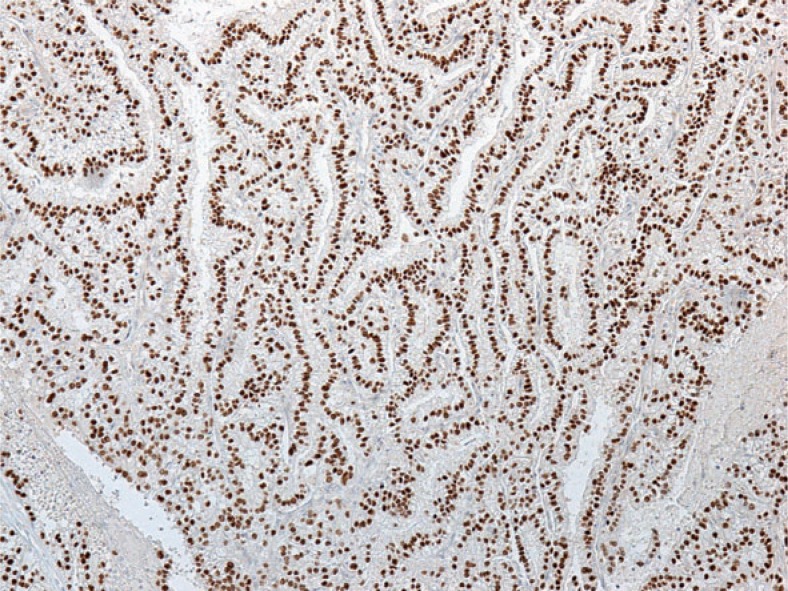
Strong diffuse nuclear transcription factor for immunoglobulin heavy-chain enhancer 3 (TFE3) immunostaining.

In both cases the tumour cells showed diffuse strong nuclear immunopositivity for transcription factor for immunoglobulin heavy-chain enhancer 3 (TFE3) confirming the diagnosis of Xp11.2 translocation renal cell carcinoma (Figure 7)

## Discussion

We report the first two documented cases of Xp11.2 translocation/TFE3 fusion renal cell carcinomas in Slovenia in young adult males with a long and unusual history. This is a rare subtype of RCC that we were probably not sufficiently aware of since it was included in the WHO classification of RCC for the first time in 2004. Its incidence is higher in children and young adults.^9^,^10^

Morphologically Xp11.2 translocation RCCs are quite heterogeneous. The most consistent histologic appearance is a carcinoma with mixed papillary and nested/alveolar architecture, composed of cells with clear and/or eosinophilic, granular, voluminous cytoplasm, discrete borders and the presence of extensive psammoma bodies. These features are partially consistent with clear cell RCC and partially with papillary RCC, therefore we believe, that in the past these tumour were misdiagnosed as one of this more common subtypes of RCC. The diagnosis of Xp11.2 translocation RCC is suspected based on clinical information, histology and immunochemical features, however, it is confirmed by the detection of chromosome translocation involving TFE3 gene at Xp11.2 using different methods.^13^ In this regard nuclear immunoreactivity for TFE3 protein cells arranged mostly papillary and focally in solid/alveolar patterns. The tumour cells were large with sharply defined borders and mostly clear, in some areas they had finely granular eosinophilic cytoplasm. Fibrovascular cords in some areas of the papillae were strongly hyalinised. Psammomatous calcifications were present in the stroma and in the capsule surrounding the tumour nodules. There by routine immunohistochemistry is a highly sensitive and specific marker.^14^,^15^ The anti-TFE3 antibody has only recently become available in our pathology department.

The biologic behaviour of Xp11.2 translocation RCC and its response to treatment is still not well defined. As described in literature, Xp11.2 translocation renal cell carcinomas are usually large tumours with focal cystic areas, haemorrhage and necrosis that present in advanced stage.^16^ Nevertheless, the prognosis in young children seems to be rather good, while in adults it seems to behave in more aggressive fashion. Prior exposure to chemotherapy is the only known risk factor for the development of these tumours.^17^ But, neither of our patients had a history of previous chemotherapy. Both of them were young adults, and presented with long-term history of complaints, so it is possible that the tumours had developed when they were younger or even in childhood, but had not been detected until reaching the dimension of a large mass or an advanced stage. Another possibility is that the Xp11.2 translocation renal cell carcinoma is more aggressive when it occurs in adults than when it occurs in children.^18^,^19^

In our cases late diagnosis was established on one hand because the diagnosis of Xp11 translocation RCC may have been previously underestimated in young adults. Initial symptoms were not clear and diagnostic evaluation and imagines were underestimated too. Cysts, haematomas, necrotic tissue in or around the kidney or even extensive psammomatous calcification evident radiographically can be misinterpreted as some traumatic injury or harmless developmental abnormality in young adults. Therefore, it is necessary to diagnose this tumour entity accurately taking into consideration all available diagnostic tools to clarify unusual pains and problems in young adults.

## Conclusions

Because of the small number of Xp11.2 translocation renal cell carcinoma described in the literature, the exact biologic behaviour and impact of current treatment modalities remain to be uncertain. Increased awareness among urologists, pathologists, and oncologist is necessary in order to help identifying more cases of this phenotype in the future. Treatment is primarily surgical; in advanced-stage target agents should be a treatment of choice but with doubtful success. When dealing with younger patients we must be aware that haematomas, cysts or necrotic tissue in/around the kidneys could represent an initial stage of translocation renal cell carcinoma. In such cases we must use appropriate imaging studies to exclude reliably malignant tumour or angimyolipoma. Lesion biopsy and the use of antibodies against TFE3 in all RCC, with emphasis on young adults, may be necessary to determine the biologic nature and incidence of this tumour.

## Figures and Tables

**TABLE 1.** Classification of renal cell tumours (adapted from EAU guidelines 2013)

Stage I, T1N0M0	Tumour is < 7 cm, confined to kidney
Stage II, T2N0M0	Tumour is > 7 cm, confined to kidney
Stage III, T1-3N0-1M0	Tumour of any size, growing into major vein, into tissue around the kidney not beyond Gerota’s fascia, spread to lymph nodes
Stage IV, T4N1-2,M1	Tumour of any size, growing beyond Gerota’s fascia, spread to nearby or distant lymph nodes, spread to organs (bones, lungs, liver)
